# Combined treatment with Nd:YAG laser and polidocanol foam sclerotherapy in venous malformation of the hand: case report

**DOI:** 10.1590/1677-5449.202501902

**Published:** 2026-07-27

**Authors:** Helena de Oliveira Santos, Genaro Fahrnholz Buonsante, Marina Araujo Zulchner, Marcos Arêas Marques

**Affiliations:** 1 Universidade Federal do Estado do Rio de Janeiro – UNIRIO, Hospital Universitário Gaffrée e Guinle, Rio de Janeiro, RJ, Brasil.; 2 Universidade do Estado do Rio de Janeiro – UERJ, Rio de Janeiro, RJ, Brasil.

**Keywords:** vascular malformations, sclerotherapy, polidocanol, Nd:YAG laser, hand, rare disease

## Abstract

Venous malformations of the hand represent a clinical and therapeutic challenge due to anatomical complexity and functional and aesthetic impact. We report the case of a 13-year-old female patient with a venous malformation of the hand, treated with a combined approach using Nd:YAG 1064 nm transdermal laser and 1% polidocanol foam sclerotherapy. The combination of photocoagulation and sclerotherapy proved safe and effective in a superficial and localized lesion. This case underscores the relevance of combined therapy as a minimally invasive alternative for venous malformations of the hand, highlighting the importance of individualized treatment according to lesion location, depth, and extent.

## INTRODUCTION

Vascular malformations are part of a heterogeneous group of lesions resulting from failures in vascular embryogenesis. They are classified according to the International Society for the Study of Vascular Anomalies (ISSVA) and are distinguished from vascular tumors by the absence of endothelial proliferation, growth proportional to the patient, and lack of spontaneous regression. They may be further divided into high-flow or low-flow lesions.^1,2^ Among low-flow lesions, venous malformations (VMs) are the most prevalent and may affect various anatomical sites, including the hand and forearm, where they represent a particular challenge due to functional and aesthetic complexity.^2^

From a pathophysiological perspective, VMs result from the presence of a dysplastic, dilated, and nonfunctional vascular network. Clinical presentation depends on the extent and location of the lesions, and may include edema, chronic pain, motor and sensory impairment, as well as local thromboembolic phenomena and inflammation.^3^ Clinically, they usually present as soft, compressible lesions with a bluish coloration and absence of thrill, increasing when the limb is in a dependent position and decreasing with elevation.^4^

Use of the current ISSVA classification, which distinguishes VMs among low-flow lesions, is important to define prognosis and therapeutic approach. Whereas capillary malformations are superficial and may be treated with transdermal laser alone, VMs may infiltrate deep planes and require combined therapies, including sclerotherapy, laser, surgery, and/or embolization.^2,5^ Although these modalities have been used separately, no reports were found describing treatment of VMs of the hand using the combination of 1064 nm Nd:YAG laser and polidocanol foam, making this an underexplored option for less invasive treatment.

## CASE REPORT

A 13-year-old female patient presented with a complaint of a bluish tumoral lesion in the right thenar region, with several years of progression, causing major functional discomfort (difficulty closing the hand), in addition to social distress.

A family member reported having noticed small bluish lesions in this region during childhood, with meaningful growth after the onset of puberty. The patient denied a history of local trauma or other associated complaints. She denied comorbidities and previous treatments as well.

**Figure 1:**
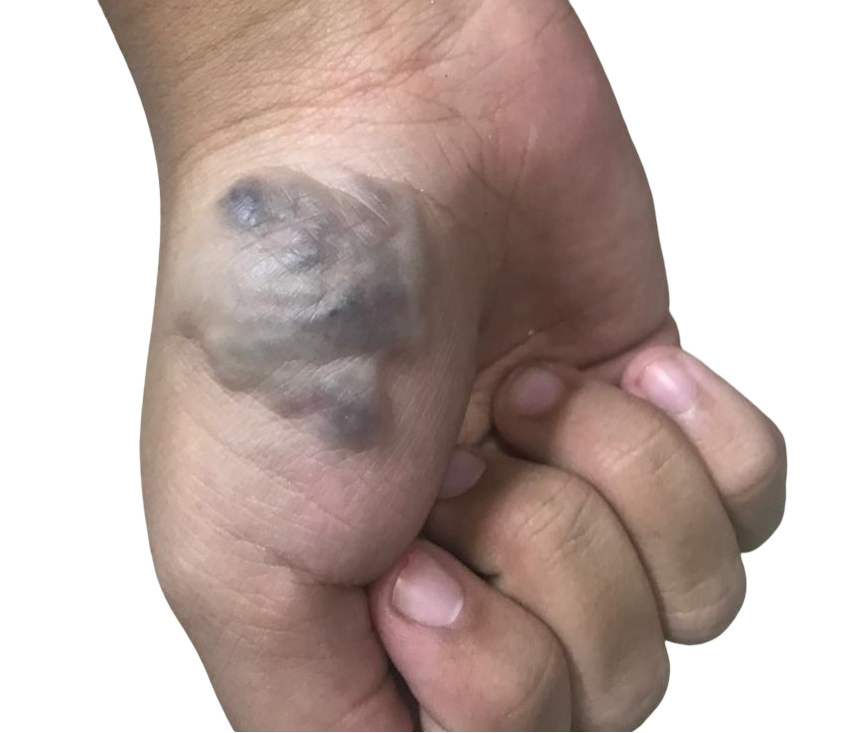
Initial presentation of lesion.

The patient sought medical care, and a bluish, soft, compressible lesion was identified in the right thenar region, without thrill or ulceration (Figure 1). Color Doppler ultrasound (CDU) examination identified a poorly defined, fully compressible lesion, with flow detected only during compressive maneuvers, measuring 2.5 × 3.0 × 2.0 cm, restricted to the skin and subcutaneous tissue, with no infiltration of the adjacent musculature, findings consistent with a low-flow venous vascular malformation according to the ISSVA classification. Given the size and characteristics of the lesion on CDU, magnetic resonance angiography was not performed.

Considering the location and superficial nature of the lesion, combined treatment with transdermal laser and foam sclerotherapy was selected. Three combined sessions of photocoagulation using a 1064 nm Nd:YAG laser on the Etherea-MX**^®^** platform (Vydence® Medical, São Carlos, Brazil) were performed, with a 6 mm spot size, fluence of 50 J/cm^2^, and pulse duration of 30 ms, followed by sclerotherapy with 1% polidocanol foam prepared using the Tessari method, with 5 mL injected in each session.

**Figure 2:**
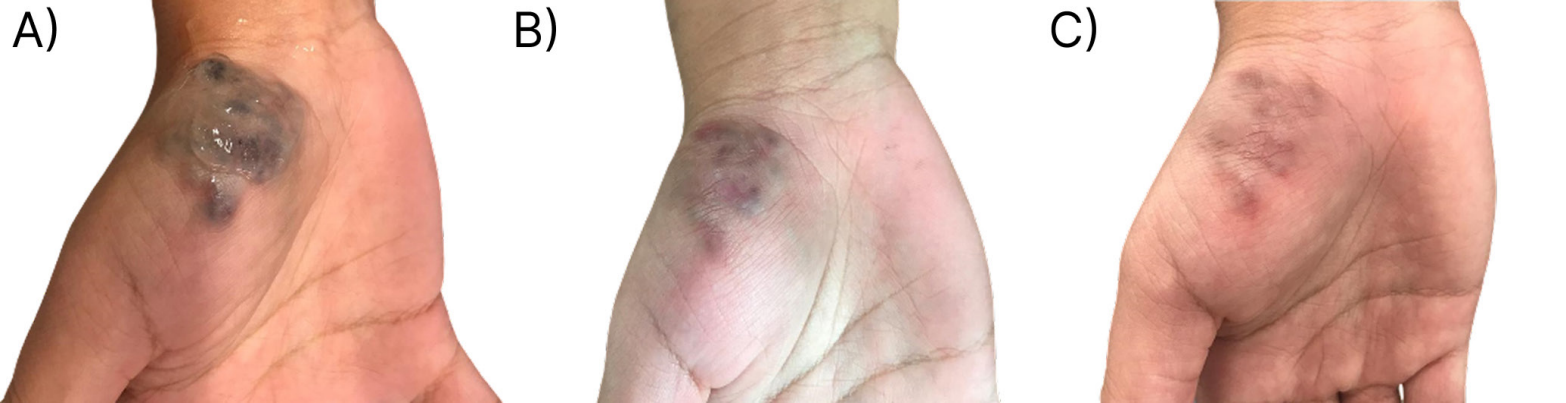
Results after the (A) first, (B) second, and (C) third sessions.

The interval between sessions ranged from 30 to 45 days, and drainage of thrombi was required during follow-up visits. Treatment was completed without additional complications, with satisfactory aesthetic and functional outcomes (Figure 2). The long-term outcome could not be assessed because the patient was lost to follow-up after the last session. This case report was approved by the research ethics committee under CAAE No. 92198125.1.0000.5244 (consolidated opinion number 7.880.663).

## DISCUSSION

Although the patient’s clinical presentation was sufficient for the diagnosis of VM, complementary examinations play an important role in the diagnosis and therapeutic planning of VMs, especially before invasive interventions.

CDU is useful in the initial assessment and, although technically difficult to perform in small or thrombosed lesions, visualization of a heterogeneous, hypoechoic, compressible mass is suggestive of VM. Magnetic resonance angiography is an option for determining the extent of the VM and its relationship with adjacent structures, demonstrating T2-hyperintense and T1-isointense lesions.^2,6^

From a therapeutic perspective, different strategies are available. Compression therapy is minimally invasive and may reduce pain and edema, but its efficacy is limited and based on low-quality studies.^7^ Surgical treatment may be indicated for localized and resectable lesions, but it is associated with risk of recurrence, functional morbidity, and technical difficulty when the malformation is diffuse or close to critical structures.^8^ Preoperative embolization may assist in extensive cases by reducing bleeding and facilitating resection, especially in lesions in which fistulous components coexist.^9,10^

CDU-guided sclerotherapy is considered a safe and effective option for the treatment of VMs of the hand, with high rates of partial and complete response.^3^ Different sclerosing agents have been described in the literature. Ethanol is highly effective but is associated with a higher risk of local complications, such as necrosis and nerve injury.^10^ Polidocanol foam seems to have a more favorable safety profile and is used at concentrations of 2% to 3%, with a maximum volume of 10 mL per session and intervals of 1 to 3 months between sessions, with a mean of 2.8 sessions per patient.^3,5^

In a meta-analysis, Horbach et al.^1^ described bleomycin, a cytotoxic agent, as having efficacy comparable to that of ethanol and sodium morrhuate in treating VMs, but with lower complication rates. Guevara et al.^11^ also suggested that sclerotherapy using sodium tetradecyl sulfate, either in association with ethanol or not, may be safely applied with good functional outcomes, even in diffuse and infiltrative malformations of the hand.

The association of transdermal 1064 nm Nd:YAG laser and polidocanol foam sclerotherapy, as used in this case report, represents an interesting strategy for the treatment of more superficial and localized lesions. This combination, initially described by Moreno-Moraga et al.^12^ for the treatment of lower-limb varicose veins, suggests greater therapeutic efficacy and, although the mechanisms involved are not fully understood, it has been hypothesized that greater endothelial damage occurs through the direct action of polidocanol and increased laser dispersion promoted by the foam. The technique allows reduction of polidocanol concentration and, consequently, its toxicity. One drawback is the increased cost of treatment due to the need for laser use.

Although no reports of combined treatment of VMs of the hand were found, use of this approach in other anatomical sites suggests that it may optimize aesthetic outcomes and reduce the need for more invasive procedures.^13,14^ The present case report describes an innovative approach for a lesion in a challenging anatomical site, with satisfactory aesthetic and functional outcomes. However, the benefit of laser associated with sclerotherapy for the treatment of VMs is still not well established in the literature and is based primarily on case reports. Ultrasound-guided polidocanol sclerotherapy alone remains a highly effective therapeutic alternative, both in terms of outcomes and cost. In addition, it should be considered that treatment with 1064 nm Nd:YAG laser is not free from complications, such as burns and nerve injury.

It is important to emphasize that, although most adverse effects of sclerotherapy are mild, such as pain and localized skin necrosis, severe cases of extensive limb necrosis following inadvertent leakage of the sclerosant into arterial vessels have been described.^15^ This risk highlights the need for the procedure to be performed in specialized centers, by an experienced multidisciplinary team.

Thus, this case report exemplifies the successful management of a VM of the hand using a minimally invasive approach, balancing aesthetic control and preservation of function, and underscores the importance of individualized therapy guided by clinical experience and scientific evidence.

## CONCLUSION

VMs of the hand constitute a diagnostic and therapeutic challenge due to the local anatomical complexity and the associated functional and aesthetic impact. The combination of transdermal 1064 nm Nd:YAG laser and polidocanol foam sclerotherapy proved to be safe and effective in the present case; however, as this is a case report, further evidence is required before this technique can be extrapolated to routine use.
