# Supplementary material for: Combined treatment with Nd:YAG laser and polidocanol foam sclerotherapy in venous malformation of the hand: case report
Source: J Vasc Bras. 2026 Jul 27;25:e20250190. doi: 10.1590/1677-5449.202501902 (PMC13446002; doi:10.1590/1677-5449.202501902)
Supplement: Translation

# Translation: Tratamento combinado com laser Nd:YAG e escleroterapia com espuma de polidocanol em malformação venosa da mão: relato de caso

**DOI:** 10.1590/1677-5449.202501901

**Authors:** Helena de Oliveira Santos, Genaro Fahrnholz Buonsante, Marina Araujo Zulchner, Marcos Arêas Marques

**Affiliations:** 1 Universidade Federal do Estado do Rio de Janeiro – UNIRIO, Hospital Universitário Gaffrée e Guinle, Rio de Janeiro, RJ, Brasil.; 2 Universidade do Estado do Rio de Janeiro – UERJ, Rio de Janeiro, RJ, Brasil.

**Keywords:** malformações vasculares, escleroterapia, polidocanol, laser Nd:YAG, mão, doenças raras

## Abstract

As malformações venosas da mão representam um desafio clínico e terapêutico devido à complexidade anatômica e ao impacto funcional e estético. Relatamos o caso de uma paciente de 13 anos de idade, sexo feminino, portadora de malformação venosa da mão, tratada com abordagem combinada de laser transdérmico Nd:YAG 1064 nm e escleroterapia com espuma de polidocanol a 1%. A associação entre fotocoagulação e escleroterapia mostrou-se eficaz e segura em lesão superficial e localizada. O presente relato reforça a relevância da terapêutica combinada como alternativa minimamente invasiva para malformações venosas da mão, destacando a importância da individualização do tratamento conforme a localização, profundidade e extensão da lesão.

## INTRODUÇÃO

As malformações vasculares são parte de um grupo heterogêneo de lesões decorrentes de falhas na embriogênese vascular. São classificadas de acordo com a Sociedade Internacional para o Estudo de Anomalias Vasculares (ISSVA), sendo diferenciadas de tumores vasculares por não apresentarem proliferação endotelial e terem crescimento proporcional ao do paciente e ausência de regressão espontânea, podendo ser ainda divididas entre lesões de alto ou baixo fluxo^1,2^. Entre as formas de baixo fluxo, as malformações venosas (MVs) são as mais prevalentes e podem acometer diferentes regiões anatômicas, incluindo a mão e o antebraço, onde representam um desafio especial devido à complexidade funcional e estética^2^.

Do ponto de vista fisiopatológico, as MVs resultam da presença de uma rede vascular displásica, dilatada e não funcional. A apresentação clínica depende do local e do tamanho das lesões, que podem incluir edema, dor crônica, alteração motora e sensorial, além de fenômenos tromboembólicos locais e inflamação^3^. Clinicamente costumam ter consistência macia e compressível, coloração azulada e ausência de frêmito, aumentar na posição pendente do membro e se reduzir com sua elevação^4^.

O uso da classificação atual proposta pela ISSVA, distinguindo as MVs dentre as lesões de baixo fluxo, é importante para a definição do prognóstico e da abordagem terapêutica. Enquanto as malformações capilares são superficiais e têm como opção o tratamento com laser transdérmico isolado, as MVs podem infiltrar planos profundos e necessitar de terapias combinadas, incluindo escleroterapia, laser, cirurgia e/ou embolizações^2,5^. Embora essas modalidades sejam utilizadas de forma isolada, não foram encontrados relatos de tratamento de MVs da mão utilizando a associação de laser Nd:YAG 1064 nm e espuma de polidocanol, permanecendo essa uma opção pouco explorada para um tratamento menos invasivo.

## RELATO DE CASO

Paciente de 13 anos de idade, do sexo feminino, com queixa de lesão tumoral azulada em região tenar direita, com vários anos de evolução, causando desconforto funcional importante (dificuldade em fechar a mão), além de transtorno social.

Uma familiar relata ter notado pequenas lesões azuladas nessa região ainda na infância, com crescimento importante após o início da puberdade. Nega história de trauma local ou outras queixas associadas. Nega também comorbidades ou tratamentos prévios.

Procurou atendimento médico, sendo identificada lesão de coloração azulada, macia e compressível em região tenar direita, sem frêmito ou ulceração (Figura 1). Ao exame de eco-Doppler colorido (EDC), foi identificada lesão de bordas pouco delimitadas, totalmente compressível, com fluxo apenas durante as manobras compressivas, medindo 2,5 × 3,0 × 2,0 cm, restrita à pele e ao tecido subcutâneo, sem infiltração da musculatura adjacente, achados compatíveis com malformação vascular venosa de baixo fluxo, segundo a classificação da ISSVA. Pelo tamanho e pelas características da lesão no ECD, optou-se por não realizar angioressonância.

**Figura 1:** Apresentação inicial da lesão.

Dada a localização e a superficialidade da lesão, optou-se por tratamento combinado com laser transdérmico e escleroterapia com espuma. Foram realizadas três sessões combinadas de fotocoagulação utilizando laser Nd:YAG 1064 nm na plataforma Etherea-MX**^®^** (Vydence® Medical, São Carlos, Brasil), com *spot* de 6 mm, fluência de 50 J/cm^2^ e duração de pulso de 30 ms, seguidas de escleroterapia com espuma de polidocanol a 1%, preparada pela técnica de Tessari, sendo injetados 5 mL em cada sessão.

O intervalo entre as sessões foi de 30 a 45 dias, sendo necessária a drenagem de trombos durante as revisões. O tratamento transcorreu sem intercorrências adicionais, com resultado estético e funcional satisfatório (Figura 2). O resultado em longo prazo não pôde ser avaliado devido à perda de seguimento da paciente após a última sessão. O presente relato foi aprovado pelo comitê de ética e pesquisa, com CAAE n^o^ 92198125.1.0000.5244 (parecer consubstanciado nº 7.880.663).

**Figura 2:** Resultados após (A) primeira, (B) segunda e (C) terceira sessões.

## DISCUSSÃO

Embora a apresentação clínica da paciente tenha sido suficiente para o diagnóstico de MV, exames complementares têm um papel importante no diagnóstico e no planejamento terapêutico das MVs, sobretudo antes de intervenções invasivas.

O EDC é útil na avaliação inicial e, embora seja tecnicamente difícil de realizar em lesões pequenas ou com trombose, a visualização de uma massa heterogênea, hipoecoica e compressível é sugestiva de MV. A angioressonância magnética é uma opção para delimitar a extensão da MV e sua relação com estruturas adjacentes, evidenciando lesões hiperintensas em T2 e isointensas em T1^2,6^.

Do ponto de vista terapêutico, diferentes estratégias estão disponíveis. A terapia compressiva é pouco invasiva e pode reduzir a dor e o edema, mas sua eficácia é limitada e baseada em estudos de baixa qualidade^7^. O tratamento cirúrgico pode ser indicado em lesões localizadas e ressecáveis, mas está associado ao risco de recidiva, morbidade funcional e dificuldade técnica quando a malformação é difusa ou próxima a estruturas críticas^8^. A embolização pré-operatória pode auxiliar em casos extensos, reduzindo o sangramento e facilitando a ressecção, principalmente em lesões nas quais coexistem componentes fistulares^9,10^.

A escleroterapia guiada por EDC é considerada uma opção segura e eficaz no tratamento de MVs da mão, com altas taxas de resposta parcial e total^3^. Diferentes agentes esclerosantes são descritos na literatura. O etanol é altamente eficaz, mas associado a maior risco de complicações locais, como necrose e lesão nervosa^10^. A espuma de polidocanol parece apresentar um perfil de segurança mais favorável, sendo utilizado em concentrações de 2 a 3%, com volume máximo de 10 mL por sessão e intervalo de 1 a 3 meses entre as sessões, com média de 2,8 sessões por paciente^3,5^.

Em uma metanálise, Horbach et al.^1^ citam a bleomicina, um agente citotóxico, como tendo eficácia comparável à do etanol e do morruato de sódio no tratamento de MVs, porém com menores taxas de complicações. Guevara et al.^11^ também sugerem que a escleroterapia utilizando tetradecil sulfato de sódio, associado ou não ao etanol, pode ser aplicada com segurança e bons resultados funcionais, mesmo em malformações difusas e infiltrativas da mão.

A associação entre laser transdérmico Nd:YAG 1064 nm e escleroterapia com espuma de polidocanol, como utilizada neste relato, representa uma estratégia interessante para o tratamento de lesões mais superficiais e localizadas. Essa associação, descrita inicialmente por Moreno-Moraga et al.^12^ para o tratamento de veias varicosas de membros inferiores, sugere maior eficácia terapêutica e, embora os mecanismos envolvidos não estejam completamente elucidados, levanta-se a hipótese de maior dano endotelial pela ação direta do polidocanol e pelo aumento da dispersão do laser promovido pela espuma. A técnica permite reduzir a concentração de polidocanol e, consequentemente, sua toxicidade. Uma desvantagem é o aumento no custo do tratamento devido à necessidade de uso do laser.

Embora não tenham sido encontrados relatos de tratamento combinado de MVs da mão, o uso dessa abordagem em outras localizações sugere que ela pode otimizar o resultado estético e reduzir a necessidade de procedimentos mais invasivos^13,14^. O presente relato de caso apresenta uma abordagem inovadora para uma lesão em localização anatômica desafiadora, com resultado estético e funcional satisfatório. Entretanto, o benefício do laser associado à escleroterapia para o tratamento de MVs ainda não está bem estabelecido na literatura, baseando-se principalmente em relatos de caso. A escleroterapia com polidocanol guiada por ultrassonografia, de forma isolada, permanece como uma alternativa terapêutica altamente eficaz, tanto do ponto de vista dos resultados quanto dos custos. Além disso, deve-se considerar que o tratamento com laser Nd:YAG 1064 nm não é isento de complicações, como queimaduras e lesões nervosas.

É importante ressaltar que, embora a maioria dos efeitos adversos da escleroterapia seja leve, como dor e necrose cutânea localizada, foram descritos casos graves de necrose extensa de membro após extravasamento inadvertido do esclerosante em vasos arteriais^15^. Tal risco evidencia a necessidade de realização do procedimento em centros especializados, por uma equipe multidisciplinar experiente.

Assim, o caso relatado exemplifica o manejo bem-sucedido de uma MV da mão com abordagem minimamente invasiva, equilibrando controle estético e preservação funcional, e reforça a importância da individualização terapêutica guiada pela experiência clínica e pelas evidências científicas.

## CONCLUSÃO

As MVs da mão constituem um desafio diagnóstico e terapêutico em razão da complexidade anatômica local e do impacto funcional e estético associado. A combinação de laser transdérmico Nd:YAG 1064 nm e escleroterapia com espuma de polidocanol demonstrou-se segura e eficaz no caso apresentando, mas por se tratar de um relato de caso, mais evidências são necessárias antes que possamos extrapolar essa técnica para uso rotineiro.
